# Long-Term Deficits in Cortical Circuit Function after Asphyxial Cardiac Arrest and Resuscitation in Developing Rats

**DOI:** 10.1523/ENEURO.0319-16.2017

**Published:** 2017-06-30

**Authors:** Jason W. Middleton, Daniel J. Simons, Jennifer W. Simmons, Robert S.B. Clark, Patrick M. Kochanek, Michael Shoykhet

**Affiliations:** 1 Department of Cell Biology and Anatomy, Neuroscience Center of Excellence, Louisiana State University Health Sciences Center, New Orleans, LA 70112; 2 Department of Neurobiology, University of Pittsburgh School of Medicine, Pittsburgh, PA 15261; 3 Department of Pediatrics, Washington University in St. Louis School of Medicine, St. Louis, MO 63110; 4 Department of Critical Care Medicine, University of Pittsburgh School of Medicine, Pittsburgh, PA 15261; 5 Safar Center for Resuscitation Research, University of Pittsburgh, Pittsburgh, PA 15260

**Keywords:** Cortex, hypoxia, ischemia, somatosensory, synchrony, thalamus

## Abstract

Cardiac arrest is a common cause of global hypoxic-ischemic brain injury. Poor neurologic outcome among cardiac arrest survivors results not only from direct cellular injury but also from subsequent long-term dysfunction of neuronal circuits. Here, we investigated the long-term impact of cardiac arrest during development on the function of cortical layer IV (L4) barrel circuits in the rat primary somatosensory cortex. We used multielectrode single-neuron recordings to examine responses of presumed excitatory L4 barrel neurons to controlled whisker stimuli in adult (8 ± 2-mo-old) rats that had undergone 9 min of asphyxial cardiac arrest and resuscitation during the third postnatal week. Results indicate that responses to deflections of the topographically appropriate principal whisker (PW) are smaller in magnitude in cardiac arrest survivors than in control rats. Responses to adjacent whisker (AW) deflections are similar in magnitude between the two groups. Because of a disproportionate decrease in PW-evoked responses, receptive fields of L4 barrel neurons are less spatially focused in cardiac arrest survivors than in control rats. In addition, spiking activity among L4 barrel neurons is more correlated in cardiac arrest survivors than in controls. Computational modeling demonstrates that experimentally observed disruptions in barrel circuit function after cardiac arrest can emerge from a balanced increase in background excitatory and inhibitory conductances in L4 neurons. Experimental and modeling data together suggest that after a hypoxic-ischemic insult, cortical sensory circuits are less responsive and less spatially tuned. Modulation of these deficits may represent a therapeutic approach to improving neurologic outcome after cardiac arrest.

## Significance Statement

Cardiac arrest survivors often have severe neurologic injury. Neurologic injury and subsequent behavioral deficits likely arise not only from arrest-related cell death but also from long-term dysfunction of neuronal circuits. We show in a rat model of pediatric asphyxial cardiac arrest that deficits in sensory information processing persist in cortical layer IV circuits for months after injury. As a general feature, hypoxic-ischemic brain injury leads to less responsive and less spatially tuned sensory cortical circuits. Understanding the mechanisms underlying abnormal circuit function after cardiac arrest may lead to new approaches for modulating neuronal circuits and restoring normal function in survivors.

## Introduction

Cardiac arrest (CA) occurs when all blood flow to the body, including to the brain, ceases. Annually in the United States, CA affects ∼350,000 adults (CDC, 2002; Roger et al., 2012) and ∼10,000 children (Atkins et al., 2009). In adults, CA occurs most commonly as a result of cardiac ischemia and subsequent arrhythmia. In children, CA often follows respiratory compromise and resulting hypoxia/asphyxia. Regardless of the etiology, survival after CA critically depends on timely initiation of cardiopulmonary resuscitation (CPR; Valenzuela et al., 1997; Karch et al., 1998; Gottschalk et al., 2002; De Maio et al., 2003; Weisfeldt et al., 2011). Rescuers in well-developed emergency response systems (ERSs) arrive and initiate CPR 6–8 min after ERS activation (Bernard et al., 2002, 2010; The Hypothermia after Cardiac Arrest Study Group, 2002; Deasy et al., 2010; Aufderheide et al., 2011; Zive et al., 2011; Wang et al., 2012). Despite such short response times, <10% of CA victims recover good neurologic function (Graves et al., 1997; Aufderheide et al., 2011; Mateen et al., 2011). Even in witnessed in-hospital pediatric CA with immediate onset of CPR and return of spontaneous circulation within 15 min, only 30% of victims survive with a good neurologic outcome (Matos et al., 2013). Thus, timely CPR restores blood flow, but resulting global hypoxic-ischemic brain injury often precludes meaningful neurologic recovery.

Neuronal cell death after 10–15 min of CA cannot fully explain the extent of neurologic injury observed in survivors. In animal CA models, 9–10 min of arrest results in only modest neuronal cell death (Myers and Yamaguchi, 1977; Pulsinelli and Brierley, 1979; Kumar et al., 1988; Radovsky et al., 1997; Böttiger et al., 1998; Geocadin et al., 2000; Shoykhet et al., 2012). Nine minutes of asphyxial CA in developing rats leads to persistent dysfunction among whisker-responsive thalamocortical (TC) neurons in the ventroposterior medial nucleus (VPm) without overt cell death in VPm or among reciprocally connected inhibitory neurons in the reticular nucleus (RT; Shoykhet et al., 2012). Similarly, in neonatal animals, a moderate hypoxic-ischemic insult impairs monocular deprivation-induced circuit reorganization in the visual cortex without overt loss of inhibitory neurons (Failor et al., 2010). A 10-min-long CA produces widespread ultrastructural alterations in ribosomes, mitochondria, and endoplasmic reticulum among neurons in the hippocampus, RT, and cerebral cortex despite only a 20% cell loss in the hippocampus (Hossmann et al., 2001) and paucity of cell death in the thalamus and cortex (Radovsky et al., 1997). Finally, rats resuscitated from a 5-min-long CA demonstrate sustained neurobehavioral deficits (Schreckinger et al., 2007), although such short arrests produce minimal cell death (Radovsky et al., 1995). Why do neurologic deficits persist even when neuronal cell death is modest? We propose that long-term neuronal circuit dysfunction after injury contributes to sustained neurologic deficits in CA survivors.

Here, we characterize long-term abnormalities of cortical layer IV (L4) barrel circuits in adult rats that survived an asphyxial CA during development. L4 is the major thalamorecipient zone in the somatosensory cortex. Because other cortical layers depend on information propagated from L4, CA-associated abnormalities in L4 can have widespread adverse consequences. In this clinically realistic model of 9 minutes of asphyxial arrest and resuscitation, neuronal death in the cerebral cortex is observed only in a subset of L5 neurons (Fink et al., 2004; Shoykhet et al., 2012). On the other hand, we find circuit-level abnormalities in L4 that persist for >6 mo after arrest, suggesting long-term circuit reorganization in survivors.

## Methods

### Animals

Thirteen female Sprague-Dawley rats were used in the experiments. CA or sham intervention was conducted at postnatal day (PND) 16–18, and neurophysiologic recordings were conducted on adult rats at least 6 mo after arrest. Only female rats were used in the study because the heavy weight (>600 g) of adult male rats in captivity presents a number of logistic and technical difficulties. First, rats this large require single housing, which is not recommended by Institutional Animal Care and Use Committee and federal regulations. Second, surgical procedures in preparation for recordings (tracheostomy, arterial and venous line placements, craniotomy) become more complex because of large amounts of fat overlying surgical landmarks and result in higher surgical mortality due to excessive bleeding associated with fat dissection. Third, overweight male rats require high inflation pressures (>25–30 cm H_2_O) to maintain adequate ventilation and oxygenation during the recordings, which leads to ongoing barotrauma, results in rapid physiologic deterioration, and limits recording time. Finally, male rats under fentanyl analgesia used during the recordings fail to urinate spontaneously because of sphincter constriction. Progressive bladder distention, if not addressed surgically with a suprapubic catheter, evokes abnormal autonomic responses and further impairs pulmonary dynamics, substantially degrading recording validity and quality. For these reasons, we focused our study on female rats. Although pediatric CA occurs more commonly in males (∼65% male vs. 35% female; Moler et al., 2015), hypoxic-ischemic brain injury tends to be less severe in females (Johnston and Hagberg, 2007). Hence, the abnormalities observed in female rats in the current study may be milder than those in male rats. Animals were housed in an Association for Assessment and Accreditation of Laboratory Animal Care–certified facility on a 12-h light-dark cycle with free access to food and water. The University of Pittsburgh and Washington University in St. Louis Animal Care and Use Committees approved all experimental procedures.

### Asphyxial cardiac arrest

Female PND16-18 Sprague-Dawley rats (35–40 g) were anesthetized with 3% isoflurane/50% N_2_O/balance oxygen, endotracheally intubated with an 18-gauge angiocatheter, and mechanically ventilated with 1%–2% isoflurane/50% N_2_O/balance oxygen for surgery (Harvard Apparatus). Femoral arterial and venous catheters were placed through skin incisions under sterile conditions, and needle electrocardiogram (ECG) electrodes and needle scalp electroencephalogram (EEG) electrodes were inserted. Mean arterial blood pressure, ECG, and EEG were continuously monitored and recorded (Model 7 Polygraph, Grass Instruments). Minute ventilation was titrated to maintain normal arterial pco_2_ (∼40 ± 2 mm Hg). Vecuronium (1 mg/kg, i.v.; Sun Pharmaceutical) was administered 10 min before asphyxia to establish neuromuscular blockade. Vecuronium was chosen for its short (15- to 20-min) duration of action in rats, such that subsequent recovery is not affected by neuromuscular blockade. Two minutes before arrest, the anesthetic gas mixture was turned off, and the rat was ventilated with room air [fraction of inspired oxygen (Fio_2_) = 0.21]. This anesthetic washout was performed to reduce the confounding effects of inhaled anesthetics (Weigl et al., 2005; Statler et al., 2006; Bickler et al., 2012).

Previous pilot experiments demonstrated that electroencephalographic activity begins to recover in frequency and amplitude 2 min after discontinuation of the anesthetic mixture. At that time, the animals are beginning to emerge from general anesthesia but have yet to regain consciousness. Therefore, at the end of the 2-min-long washout period, the ventilator was turned off for 9 min. With this experimental paradigm, apnea inevitably leads to pulseless CA within 60–90 s as demonstrated by the arterial wave form. At the end of the 9-min period of asphyxia, the rats were resuscitated using a clinically realistic algorithm based on the guidelines for human Advanced Cardiac Life Support (AHA, 2006). Mechanical ventilation was restarted with 100% O_2_ (Fio_2_ = 1.0); intravenous epinephrine (0.005 mg/kg) and sodium bicarbonate (1 mEq/kg) were administered; and manual chest compressions (∼300/min) were performed until either return of spontaneous circulation (ROSC) or 2 min had elapsed from initiation of resuscitation. If rats did not attain ROSC within 2 min of resuscitation, they were killed.

After resuscitation, vascular catheters were removed, all surgical incisions were closed, and rats were weaned from mechanical ventilation. Rats that failed to separate from mechanical ventilation by 1 h after CA were killed. After extubation, rats were observed in Fio_2_ = 1.0 for 1 h to mimic a clinical scenario and then returned to their mothers. Pups were kept with their mothers until PND28 and then weaned. Sham rats underwent all procedures except asphyxia and resuscitation. Survival rate in this model of cardiac arrest is ∼85% (Fink et al., 2004), with mortality mostly occurring within the first 24–48 h owing to the animal’s poor neurologic condition. Ten rats underwent cardiac arrest, two were killed within 48 h postarrest because of inability to self-care, and eight survived long-term to undergo recordings. Mortality in the sham group is rare, such that all five rats that underwent sham surgery survived to undergo recordings. The age of the rats at the time of recordings was 8 ± 2 mo.

### Surgical preparation for neurophysiologic recordings

Rats were anesthetized with isoflurane, and a tracheal tube was inserted via tracheostomy to maintain a clear air passage. Tracheostomy is required to allow unimpeded access to the whiskers for stimulation and to allow for suctioning of secretions from the airway during the recording session. Small-diameter Silastic tubing was inserted into the external jugular vein for drug delivery, and a small Teflon catheter was inserted into the right femoral artery for monitoring blood pressure. The skull was exposed, and small stainless steel screws were inserted into the bone over the left occipital and frontal lobes for electrocorticogram (ECoG) monitoring; an additional screw was inserted into the bone over the right frontal lobe to serve as a reference for cortical microelectrode recordings. Bone overlying the right primary somatosensory cortex was thinned with a handheld microdrill. For unit recordings, a small area (<0.5 × 0.5 mm^2^) of thinned bone was removed overlying the right barrel cortex (∼3 mm posterior to bregma and ∼5 mm lateral to midline). Saline was periodically applied to an acrylic dam constructed around the craniotomy. All wound edges were infiltrated with 2% lidocaine upon completion of the surgical procedures.

During the recording session, isoflurane was discontinued, and the rat was maintained in a lightly sedated state using fentanyl (Baxter Health Care Corporation, 10 μg/kg/h). The rat was immobilized with pancuronium bromide (Sicor Pharmaceuticals, 1.6 mg/kg/h) to prevent spontaneous whisker movements that could otherwise interfere with use of our whisker stimulators (below). We used pancuronium during the recordings because of its long duration of action. Body temperature was maintained at 37°C using a servo-controlled heating blanket (Harvard Apparatus). Blood pressure, heart rate, tracheal airway pressure, and ECoG were monitored throughout the recording session with a personal computer using custom-written software. We decided *a priori* that if we could not maintain these indicators within normal physiologic ranges (mean arterial pressure >60 mm Hg, heart rate >300 bpm, peak airway pressure <30 cm H_2_O, no seizure activity, and minimal bursting on the ECoG), we would terminate the recording session. No recordings were terminated prematurely in this study.

### Whisker stimulation

Whiskers were deflected one at a time using a piezoelectric stimulator attached 10 mm from the base of the whisker (Simons, 1983). Stimulus waveforms, stored on disk, were output at 10 kHz via an eight-channel digital-to-analog converter. Whiskers were randomly deflected 1 mm in one of eight directions (0°, 45°, 90°, etc.) using a ramp-and-hold stimulus. The ramp phase of the deflection was ∼8 ms long, with a mean velocity of 125 mm/s. The whisker deflection was maintained for 200 ms, and the whisker was then returned to its resting or neutral position with the same speed as the initial deflection.

### Recording

Simultaneous multiple, single-unit recordings were obtained using a multichannel Eckhorn matrix (MM-5, Thomas Recording). Platinum/iridium in quartz fibers (60-μm diameter) were pulled and ground to 2- to 5-μm tip diameters with impedances of 1–6 MΩ. One by one, electrodes were brought into contact with the pial surface and advanced into L4 (∼700 μm below the pial surface) before positioning of the next electrode. The Eckhorn matrix and the accompanying software allow for independent movement of each electrode in the *z*-axis. Typically, three electrodes inserted into the brain 100–200 μm apart were used simultaneously in each recording session. After characterizing a single neuron on a given electrode, that electrode was advanced in four steps until a spike from a different neuron was encountered. Usually, an electrode is moved 50–100 μm from the prior recording depth to optimize wave form discrimination of the newly encountered neuron. After traversing a full depth of L4 (700–1000 μm below the pial surface), the electrode was withdrawn from the brain and repositioned 20–50 μm away from the previous recording location without disturbing the other electrodes in the array.

**Figure 1.**
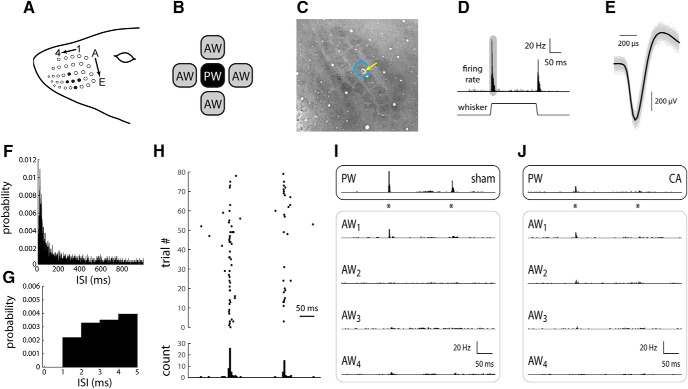
Response of L4 single RSUs in whisker/barrel cortex of sham and CA rats. ***A***, The rat whisker pad consists of an ordered array of order whiskers; as an example, filled black circles indicate whisker D3 as the PW (see Materials and Methods), whiskers D2 and D4 as AWs in the same row, and whiskers C3 and E3 as AWs in the same column. ***B***, To characterize spatial receptive field properties (relative responses across the whisker array), we recorded from neurons while stimulating the PW and two to four AWs, individually, in either the same column or same row as the PW. ***C***, An example tangential section of the L4 barrel field stained for CO (see Materials and Methods), illustrating an electrolytic lesion to confirm the location of the primary whisker. ***D***, In response to a 1-mm ramp-and-hold whisker deflection (see Materials and Methods), an L4 RSU responds with a robust, transient increase in firing rate. After the whisker deflection offset, the unit responds with a similar, albeit smaller, transient increase in firing rate. The shaded area schematically represents the time window for computing the ON response. ***E***, A representative wave form of a single-unit RS neuron recording. All recorded waveforms are shown in gray. The average wave form is shown in black. ***F***, ISI histogram for all recorded units in sham rats. The ISI histogram in CA rats is essentially identical (not shown). Bin width, 1 ms. ***G***, The first 5 ms of the ISI histogram in ***F***. Note absence of recorded spikes in the absolute refractory period of 1 ms. ***H***, The set of spike time responses of an example sham neuron, presented as a spike time raster plot, illustrates the sparseness of spontaneous activity and relative robustness of whisker evoked responses. ***I***, The peristimulus time histogram (PSTH) of a representative RSU from a sham rat in response to PW deflection (asterisks indicate onset and offset times) indicates a robust, temporally precise response (top). The responses to the four AWs are smaller in amplitude, and in at least one case (AW_2_), there is no appreciable response. ***J***, An L4 RSU from a CA rat displays a small PW-evoked response similar in magnitude to those evoked by some of the AWs (AW_1_ and AW_2_).

The principal whisker (PW) of a cortical neuron is defined as the whisker whose deflection evokes the largest spiking response relative to other whiskers (Fig. 1). The adjacent whiskers (AWs) were defined as whiskers immediately rostral, caudal, dorsal, and ventral to the PW (Fig. 1*B*). The PW corresponds anatomically to the barrel in which the recorded neuron is located (Fig. 1*C*). This relationship was later confirmed by histologic analysis. Microelectrode signals were bandpass filtered (300 Hz to 10 kHz) and passed to a personal computer, where spike waveforms were detected in real time using custom-programmed acquisition software (Labview, National Instruments) and stored for further analysis. Unless otherwise reported, means and SEs are the population means of 34 neurons recorded in five sham rats and 57 neurons recorded in eight CA rats.

### Data analysis

Action potential firing, or spiking, evoked by whisker deflections was characterized by magnitude of responses and stimulus specificity. For each neuron, response magnitudes to deflection onsets (ON) and offsets (OFF) were quantified as the average number of spikes per stimulus discharged in the appropriate 25-ms-long response window (Fig 1*D*). Receptive fields (RFs) are defined as the set of whiskers that evoke a spiking response in individual L4 neurons (Simons and Carvell, 1989). For each cell, RF focus was quantified by calculating the ratio of responses to adjacent and principal whiskers (AW/PW). Small or large AW/PW ratios indicate narrow or broad spatial focus, respectively, on the PW. An average AW value was calculated for each neuron by using the responses to different AWs for that particular neuron. Depending on the recording and whisker stimulation conditions, one to four AWs contributed to this average for each neuron.

Putative excitatory cells generate regular spike (RS) extracellular waveforms characterized by an asymmetric biphasic wave form with a broad initial negative component (200–400 μs; Fig. 1*E*; Bruno and Simons, 2002). Putative inhibitory cells generate fast spike (FS) extracellular waveforms, which are characterized by a more symmetric biphasic wave form with a fast initial negative component (100–180 μs; Bruno and Simons, 2002). This study focused specifically on RS cells for two reasons. First, FS neurons constitute ∼10% of L4 barrel neurons (Beaulieu, 1993) and hence occur less frequently in extracellular recordings. For example, in this study, we recorded four and eight FS neurons in sham and CA rats, respectively (∼1 FS neuron/rat), which is insufficient for quantitative comparisons. Second, unambiguous identification of FS neurons in extracellular recordings requires special processing of electrodes and specific filter settings, which makes simultaneous recording of FS and RS neurons challenging (Bruno and Simons, 2002). Spike waveforms were examined with cluster analysis using custom-programmed software in Labview. Recorded spike waveforms were sorted into unit clusters offline using principal component analysis in 2D space. Cut clusters were then examined to remove outlier (nonsimilar) waveforms. Interspike interval (ISI) histograms were checked for each unit to ensure absence of ISIs <1 ms, which corresponds to the absolute refractory period. Only well-isolated units with uniform action potential waveforms and interspike intervals that respect the absolute refractory period were used in the analyses (Fig. 1*E–G*). After sorting, mean spike waveforms were calculated, and the durations of early and late components of the waveforms were measured. A 2D scatterplot of these two components reveals two clusters (Bruno and Simons, 2002), and cell type identity was assigned based on this criterion. Individual, well-isolated units were recorded on different electrodes of the multielectrode array. Only one unit was taken from a single microelectrode.

Correlation of spiking activity between pairs of simultaneously recorded cells was quantified by the joint peristimulus histogram (jPSTH). The jPSTH is a 2D plot that gives the number of coincident spikes as a function of the relative spike times of the two compared neurons:

(1) $$jPSTH\left(t_{i},t_{j}\right)=\underset{k}{\sum }\delta_{1}^{k}\left(t_{i}\right)\delta_{2}^{k}\left(t_{j}\right),$$

where $\delta_{n}^{k}$ is the *k*th trial of the spike train of neuron 1 or 2. To quantify the number of coincidences above chance level, we subtract a trial-shuffled version of the jPSTH from the trial-matched version, giving the shuffle-corrected jPSTH. The correlation is computed by taking the diagonal of the shuffle-corrected jPSTH and dividing it by the product of the individual PSTHs:

(2) $$correlation\left(t\right)=\frac{jPSTH\left(t,t\right)}{PSTH_{1}\left(t\right)PSTH_{2}\left(t\right)}.$$

All data analyses were performed on whisker responses from 80 trials, 10 repetitions of each of the eight angular directions. Spike counts, PSTHs, and jPSTHs were averaged over 80 trials before being used to calculate population means.

### Histology

Upon termination of an experiment, the rat was deeply anesthetized with sodium pentobarbital (100 mg/kg, i.v.) and transcardially perfused (2% paraformaldehyde and 1.5% glutaraldehyde in 0.1 m phosphate buffer) for cytochrome oxidase (CO) histochemistry (Wong-Riley et al., 1978). The cortex was cut tangentially (60-μm sections), and sections were reacted for CO and counterstained with thionine. Using microdrive readings, signs of tissue disruption, and electrolytic lesions made during the experiment, recording sites were localized with respect to individual underlying barrels. Histologic evaluation indicated that there was no systematic difference between sham and CA rats with respect to recording locations. In an independent cohort of CA (*n* = 5) and sham (*n* = 5) rats, used for recordings elsewhere in the brain and subjected to an identical experimental paradigm, the brains were cut in a coronal plane and stained with thionine. In this cohort, cortical thickness was measured in the motor cortex and the whisker subfield of the primary somatosensory (barrel) cortex 2.6–3.0 mm posterior to bregma (Paxinos and Watson, 2007). Measurements were not corrected for shrinkage; however, all brains were processed similarly with expected shrinkage of ∼30%. Image manipulation was limited to autocontrast, autotone, and autocolor adjustments in Adobe Photoshop and was applied to the entire image.

### Computational modeling and simulations

A leaky integrate-and-fire neuron model was used to simulate the voltage dynamics and spiking activity of L4 neurons (Gabbiani and Cox, 2010). The model has the following form:

(3) $$C\frac{dV}{dt}=-g_{leak}\times \left(V-V_{leak}\right)+\mu +I_{background}+I_{whisk},$$

where *C* is the membrane capacitance, *g_leak_* and *V_leak_* are the conductance and reversal potential for the leak current, μ is the bias current, and *I_background_* and *I_whisk_* are the currents from background and whisker-driven synaptic sources, respectively. The background currents represent synaptic inputs from non–stimulus driven sources, and thus ongoing, spontaneous background fluctuating input currents. The background current follows the form

(4) $$I_{background}=-\sigma_{E}g_{E}\left(t\right)\times \left(V-V_{E}\right)-\sigma_{I}g_{I}\left(t\right)\times \left(V-V_{I}\right),$$

where σ is the maximal conductance scaling factor and *V* is the reversal potential for the background synaptic currents; the subscripts *E* and *I* refer to excitatory and inhibitory components, respectively. The time-dependent conductance, *g*(*t*), is described by the following:

(5) $$g\left(t\right)=\underset{i}{\sum }a\left(t_{i}\right).$$

The function *a*(*t*) is the time course of a unitary synaptic event at time *t_i_* according to

(6) $$a\left(t\right)=\alpha^{2}t\times e^{-\alpha t}.$$

The rates of unitary excitatory and inhibitory synaptic conductances are *r_E,background_* and *r_I,background_*, respectively. The timescale of synaptic conductances generated in this manner is given by *τ* = 1/*α*. The occurrence times of background excitatory and inhibitory synaptic events are Poisson distributed.

Similarly, the current evoked in a model L4 neuron by whisker deflection is given as a combination of thalamocortical-driven monosynaptic excitatory and TC-driven disynaptic inhibitory currents:

(7) $$I_{whisk}=-\sigma_{E,whisk}g_{E,whisk}\left(t\right)\times \left(V-V_{E}\right)-\sigma_{I,whisk}g_{I,whisk}\left(t\right)\times \left(V-V_{I}\right),$$

where the time-dependent conductances, *g*(*t*), have the same functional form as the background components.

The rate of whisker-driven cortical excitatory and inhibitory currents is given by

(8) $$r_{whisk}\left(t\right)=\left\{ \begin{array}{ll} r_{spont}, & t<t_{whisk}\\ r_{spont}+\frac{\beta_{evoked}\begin{array}{l} \left(e^{-\frac{t-t_{whisk}}{\tau_{2}}}-e^{-\frac{t-t_{whisk}}{\tau_{1}}}\right) \end{array}}{\tau_{2}-\tau_{1}}, & t\geq t_{whisk} \end{array}\right.$$

where *r_spont_* is the spontaneous firing rate, τ_1_ and τ_2_ are the time constants for the rise and fall of the time-dependent firing rate, and β*_evoked_* is the firing rate scaling factor. The unitary synaptic conductance times are Poisson distributed according to this time-varying rate.

To study output spike correlations between a pair of neurons, we defined an additional parameter, *c*, which controlled the correlation of the two input synaptic conductances. To each input conductance, independent events were added with a rate of *r*(1 – *c*) and common events with a rate of *rc*.

The chosen spike threshold values are within the range observed in the cortex (Dembrow et al., 2010; Pidoux et al., 2011). Maximal values for synaptic conductances are consistent with those used in other cortical network models (Lisman et al., 1998). These conductance values result in synaptic potentials with amplitudes <1 mV, consistent with experimentally observed amplitudes for thalamocortical (Bruno and Sakmann, 2006) and intracortical (Lefort et al., 2009) synaptic potentials. Timescales for the stimulus-evoked TC-driven monosynaptic excitation and disynaptic inhibition were set to qualitatively reproduce the relative time-varying nature of the thalamocortical excitatory and the intracortical inhibitory PSTHs (Khatri et al., 2004). The spontaneous rate of thalamocortical inputs to model L4 cortical neurons is based on experimentally observed spontaneous firing rate of VPm neurons (Simons and Carvell, 1989; Shoykhet and Simons, 2008) combined with the estimated level of thalamocortical convergence onto L4 regular spike units (RSUs; Bruno and Sakmann, 2006). The time scales of the synaptic conductances are within the range observed at thalamocortical (Higley and Contreras, 2006) and intracortical (Oswald and Reyes, 2008, 2011) synapses.

The parameters of the model, unless otherwise specified, are as follows: intrinsic parameters: *C* = 1 mF/cm^2^, *g_leak_* = 0.0375 mS/cm^2^, *V_leak_* = –80 mV, *μ* = 32 mA/cm^2^, *v_thresh_* = –40.4 mV, *v_reset_* = –80 mV; synaptic parameters: *σ_E_*= 0.22 mS/cm^2^ · s, *τ_E,background_* = 5 ms, ${\dot{V}}_{E}$ = 0 mV, *σ_I_*= 0.20 mS/cm^2^ · s, *τ_I,background_* = 20 ms, *V_I_* = –80 mV, *σ_E,whisk_*= 0.1 mS/cm^2^ · s, *τ_E,whisk_ =* 2 ms*, τ_1E_* = 0.1 ms, *τ_2E_* = 4 ms, *σ_I,whisk_*= 0.07 mS/cm^2^ · s, *τ_I,whisk_ =* 4 ms*, τ_1I_* = 0.6 ms, *τ_2I_* = 8 ms, *r_spont,E_* = 2 × 10^3^/s, *r_spont,I_* = 2.3 × 10^3^/s, β*_evoked,E_* = 4 × 10^3^, β*_evoked,I_* = 4.2 × 10^3^.

### Statistics

All statistical analyses were performed in Matlab. Data are presented as mean ± SEM. Sample size is the number of RSUs recorded in each experimental group. Recordings and analyses were performed by an experimenter blinded to injury status. *In vivo* recording data were analyzed as shown in Table 1.

**Table 1.** Statistical analyses

Compared values	Data structure	Type of test	*p* Value	Cohen’s *d*
AW/PW ratio	Not normal	Wilcoxon rank sum	<0.001	0.73
PW ON response	Not normal	Wilcoxon rank sum	<0.002	0.87
AW ON response	Not normal	Wilcoxon rank sum	0.6846	0.12
Spontaneous firing rate	Not normal	Wilcoxon rank sum	0.08	0.27
Motor cortex thickness	Normal	Student’s *t* test	0.67	0.29
Barrel cortex thickness	Normal	Student’s *t* test	0.80	0.33

## Results

We recorded responses of L4 barrel cortex neurons to whisker deflections in sham rats and rats subjected early in life to a 9-min-long CA followed by resuscitation. Single-neuron recordings of RSUs (Fig. 1*E–G*), which correspond to excitatory neurons (Simons, 1978; Bruno and Simons, 2002), located within barrel centers in L4 are included in the analyses. We analyzed data from 34 RSUs in five sham animals and 57 RSUs in eight CA rats.

### RSU receptive fields are less spatially focused in CA survivors

The rat whisker pad is comprised of an ordered array of whiskers that are identified by the row (lettered) and column (numbered) they occupy (Fig. 1*A*). L4 neurons in primary somatosensory cortex respond robustly to whisker deflection onsets and offsets (Simons, 1978). The whisker that evokes the most robust response, called the principal whisker, corresponds anatomically to the barrel in which that neuron is recorded (Fig. 1*A–D*). The whiskers immediately adjacent to the PW (AWs, shown schematically in Fig. 1*B* and represented as whiskers C3, D2, D4, and E3 surrounding the D3 PW in Fig. 1*A*) normally evoke no or much smaller response (Simons and Carvell, 1989). Fig. 1*C* shows a tangential section of the L4 barrel field in which the recording location of an RSU with a PW functionally identified as the D3 whisker is confirmed by an electrolytic lesion in the anatomically corresponding D3 barrel.

For a normal L4 RSU, spontaneous and PW-evoked firing is sparse on a single-trial basis, but the trial-averaged PSTH is nevertheless robustly temporally locked to whisker deflection (Fig. 1*H*). Example sets of responses of L4 neurons from sham (Fig.1*I*) and CA (Fig. 1*J*) rats illustrate typical differences in receptive field (RF) structure arising from CA-induced hypoxia-ischemia. In sham rats, as in normal rats (Simons and Carvell, 1989; Shoykhet et al., 2005; Shoykhet and Simons, 2008), the PW response is much larger than the AW response (Fig. 1*I*). In contrast, in CA rats, the PW response is similar in magnitude to the AW response (Fig. 1*J*).

**Figure 2.**
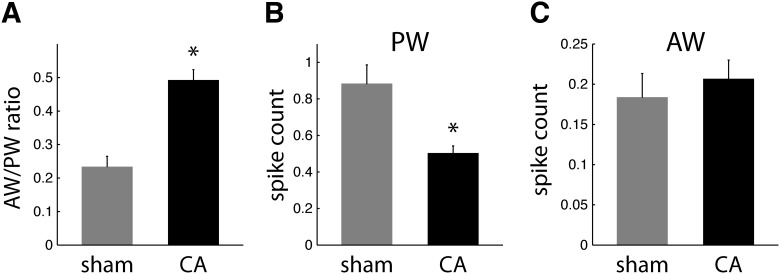
Receptive field properties of regular spike neurons in L4. ***A***, AW/PW response ratios are larger in CA neurons (0.49 ± 0.03; *n* = 57) than in sham neurons (0.23 ± 0.03; *n* = 34; WRST, *p* = 0.0002). ***B***, Mean spike counts (observed in a 25-ms window after whisker deflection) in response to PW deflections are larger for sham neurons (0.89 ± 0.11) than for CA neurons (0.50 ± 0.04; WRST, *p* = 0.0002). ***C***, Sham and CA neurons have similar AW-evoked spike count responses (sham, 0.18 ± 0.03; CA, 0.21 ± 0.02; WRST, *p* = 0.6846).

To quantify the stimulus specificity of receptive fields among L4 neurons in sham and CA rats, we calculated a measure of RF focus as the ratio of the AW-evoked response to the PW-evoked response for each neuron (i.e., AW/PW response ratio). A smaller AW/PW indicates a more narrowly focused RF, i.e., an RF more spatially focused on the PW. Conversely, a larger AW/PW indicates a more broadly focused RF, i.e., an RF less spatially focused on the PW. Hence, we compared the AW/PW ratios of L4 RSUs between CA and sham rats. The mean AW/PW of RS neurons in CA rats increased twofold compared with sham rats [0.49 ± 0.03 vs. 0.23 ± 0.03; Wilcoxon rank sum test (WRST), *p* < 0.001; Fig. 2*A*]. This finding suggests that RS neurons have broader, less PW-specific receptive fields several months after CA.

The difference in AW/PW ratios can arise from differences in PW responses, differences in AW responses, or a combination of both. We found that PW-evoked responses of RS neurons are reduced by 44% in CA rats compared with sham rats (CA, 0.50 ± 0.04 spikes; sham, 0.89 ± 0.11 spikes; WRST, *p* < 0.002; Fig. 2*B*). In contrast, AW-evoked responses of RS neurons were similar in CA and sham rats (CA, 0.21 ± 0.02; sham, 0.18 ± 0.03; WRST, *p* = 0.6846; Fig. 2*C*). Spontaneous firing rates of L4 RSUs, calculated using the observed spike counts in the 150-ms recorded period preceding whisker stimulation, were also similar in CA and sham rats (CA, 1.6 ± 0.4 Hz; sham, 2.5 ± 0.7 Hz; WRST, *p* = 0.08). Together, these findings indicate that RSU receptive fields in CA rats broaden because of a decrease in PW-evoked responses rather than an increase in AW-evoked responses.

### Barrel cortex architecture is preserved in CA survivors

**Figure 3.**
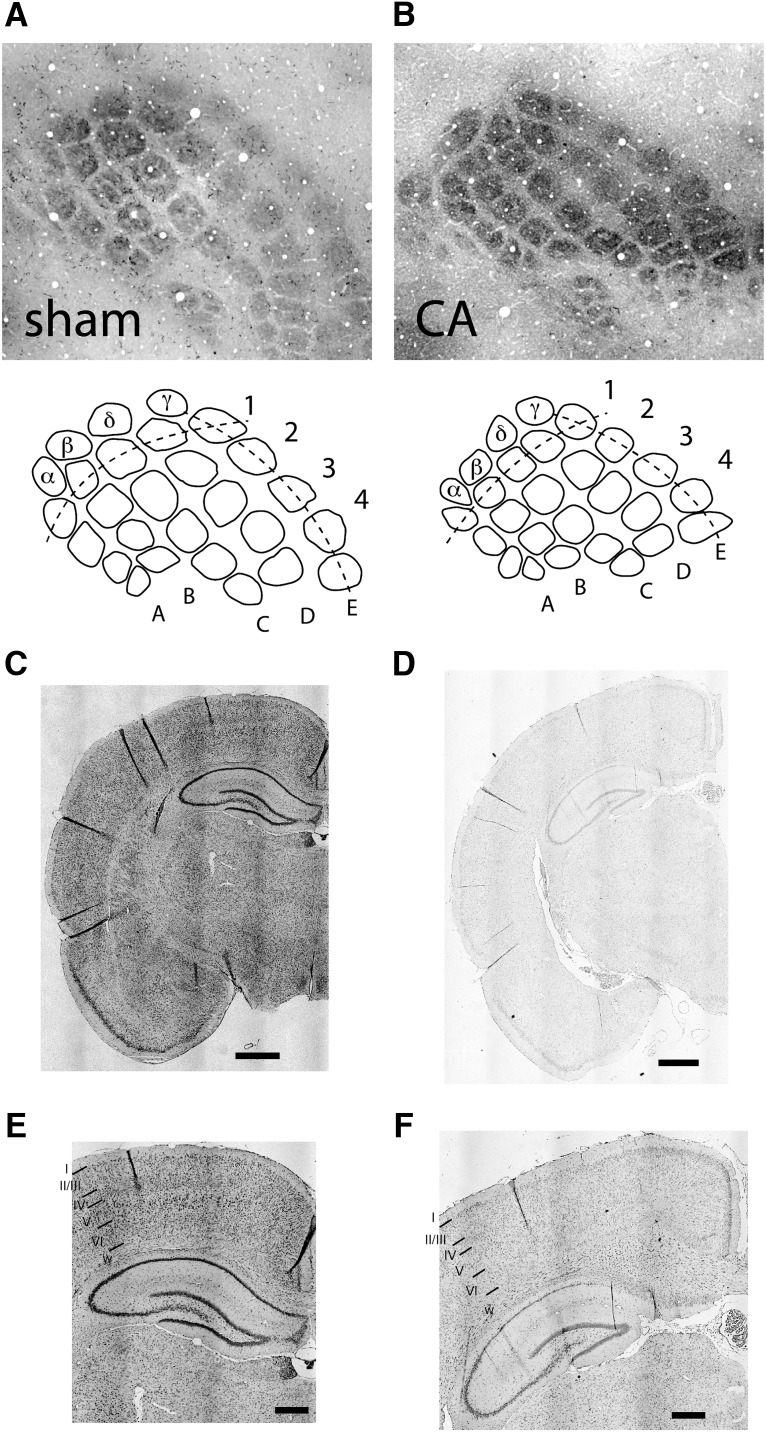
Barrel and cortical laminar cytoarchitecture in sham and CA rats. ***A***, CO histology reveals a well-ordered somatotopic map of the whisker array in L4 primary somatosensory cortex in sham rats. ***B***, No apparent gross abnormalities were observed in the barrel cytoarchitecture in CA animals. ***C***, ***D***, Normal gross brain morphology in CA rats (***D***) compared with sham rats (***C***). Nissl stain. Scale bar, 1 mm. ***E***, ***F***, Preserved cortical laminar structure in CA rats (***F***) compared with sham rats (***E***). Images enlarged from ***D*** and ***C***, respectively. Scale bar, 0.5 mm. Notice neuronal loss in the CA1 region of the hippocampus.

The whisker-responsive region of rodent somatosensory cortex consists in L4 of cytoarchitecturally distinct regions (barrels) that can be visualized with CO immunohistochemistry (Land and Simons, 1985). Barrels form during early postnatal development and are structurally stable by PND 5, with further refinement of thalamocortical projections through PND 12 (Inan and Crair, 2007). Barrel structure can be disrupted by physical injury to whisker follicle innervation before PND 5 (Van der Loos and Woolsey, 1973) and by perinatal hypoxia-ischemia at PND 3 (Quairiaux et al., 2010). In contrast, neonatal sensory deprivation without injury to peripheral receptors preserves barrel morphology (Akhtar and Land, 1991; Land et al., 1995). Our injury model induces CA relatively late in barrel morphologic development (PND 17–19), and we hypothesized that barrel fields would be grossly anatomically normal in CA rats. Indeed, there were no observable differences between sham and CA rats in the gross anatomic appearance of individual barrels or the anatomic organization of the whisker barrel field (Fig. 3*A*, *B*). Barrels in CA rats had well-defined CO-rich centers surrounded by Nissl-rich sides and were qualitatively similar in size to those in sham rats. In a separate cohort of adult CA and sham rats used for neurophysiologic recordings elsewhere in the brain, we cut the brains in a coronal plane. In CA rats, cortical laminar architecture appeared to be preserved without evidence for laminar necrosis, despite clear neuronal loss (and occasional finding of coagulative necrosis) in the hippocampal CA1 region (Fig. 3*C–F*). Cortical thickness did not differ between CA and sham rats in the motor (sham, 1.55 ± 0.10 mm; CA, 1.58 ± 0.09 mm; Student’s *t* test, *p* = 0.67) or in the primary somatosensory cortex (sham, 1.47 ± 0.04 mm; CA, 1.45 ± 0.09 mm; *p* = 0.8).

### Numerical models of sham and CA L4 neurons receiving feedforward thalamocortical inputs

**Figure 4.**
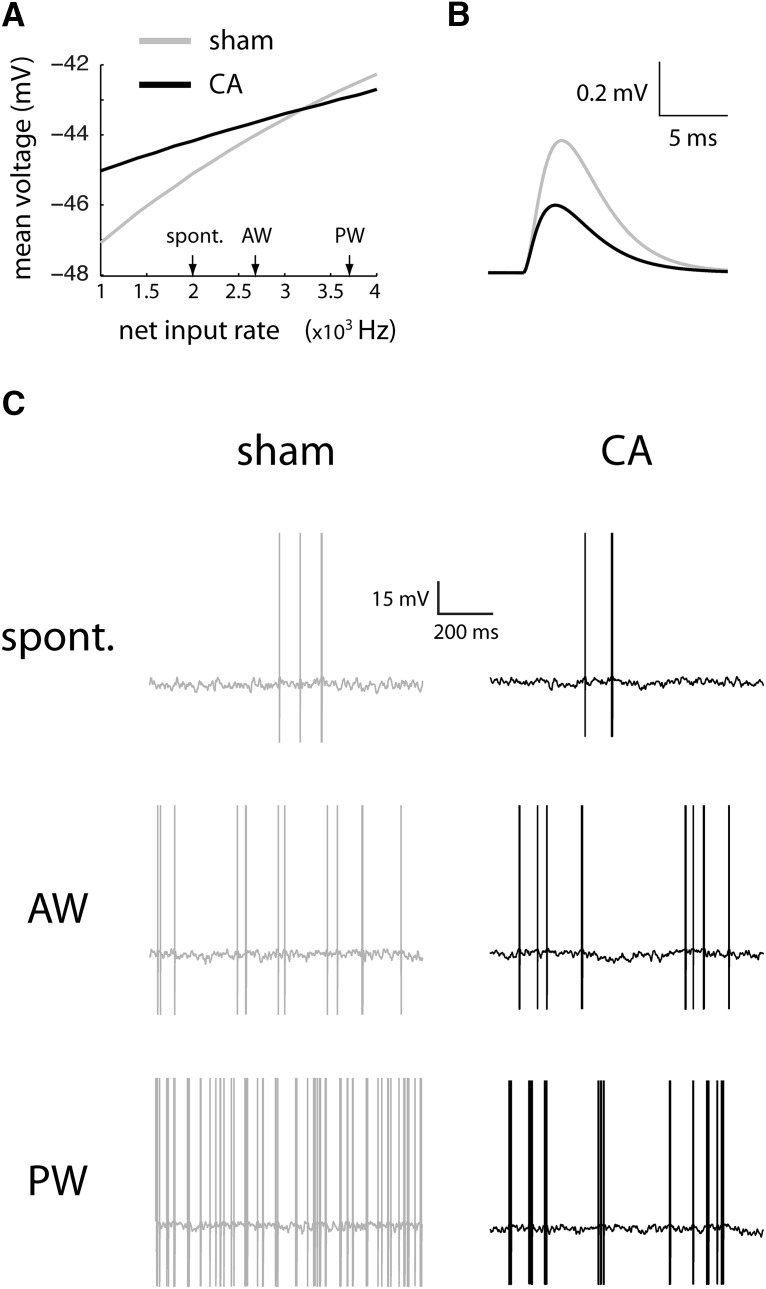
Subthreshold membrane and spiking properties of sham and CA model neurons. ***A***, When spiking threshold is removed, the mean voltage of the sham model (gray) increases with increases in input firing rate. The mean voltage for the CA model (black) starts at higher levels but increases more slowly with increasing input rate. The reduced gain of membrane voltage arises from a higher level of balanced background excitatory and inhibitory fluctuating conductances. The higher conductance shunts a higher proportion of the feedforward inputs. ***B***, Consistent with increased shunting observed for the mean depolarization, the integration of single synaptic inputs is smaller for the CA model (black) than for the sham model (gray). ***C***, Sham (left) and CA (right) models fire at comparable rates when driven with input rates corresponding to spontaneous levels (top row). Similarly, input firing rates corresponding to AW-evoked thalamic inputs result in comparable output rates in both models (middle row). However, a high level of input firing, corresponding to PW-evoked thalamic inputs (bottom row), effectively drives the sham model (left) but fails to drive the CA model to high levels (right).

**Figure 5.**
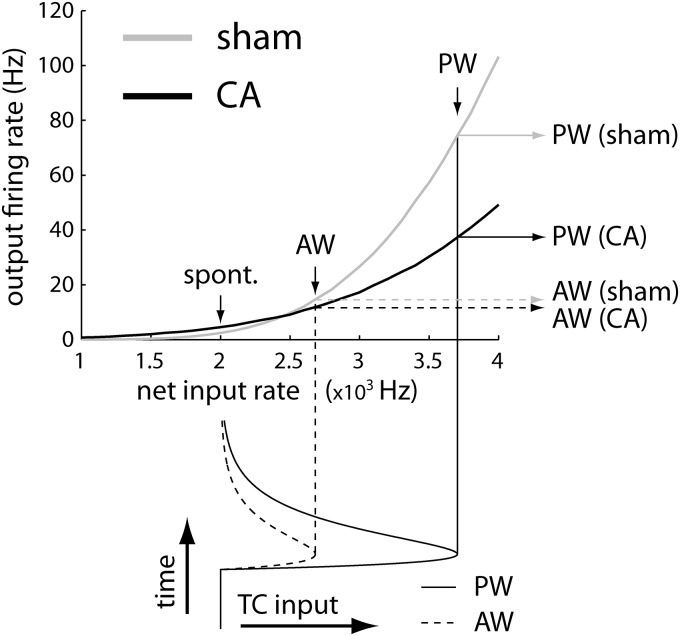
Input–output relations and AW/PW responses of L4 sham and CA model neurons. Static F-I curves for the sham model (gray) and CA model (black) neurons are calculated as a function of the firing rate of the feedforward input pathway. The lower traces show the range of instantaneous firing rates spanned by the simulated PW (solid line) and AW (dashed line) feedforward inputs in the dynamic models. At the level of spontaneous input firing rates, the output F-I curves have similar values for both sham and CA models. For peak AW input firing rates, the F-I curves for sham and CA models also have similar stationary output firing rates; the sham AW response is slightly higher than the CA AW response. In contrast, the F-I curve for the sham model is much higher at the level of peak PW input rates than the F-I curve for the CA model.

In CA survivors, broader RSU receptive fields result from smaller PW-evoked responses, although overall neuronal excitability, as measured by spontaneous firing, is unchanged. One plausible mechanism is a total increase in background conductance, affecting both excitatory and inhibitory inputs. We used a leaky integrate-and-fire model (see Materials and Methods) to test the hypothesis that the increase in conductance leads to less robust responses to PW- versus AW-evoked deflections, with little or no change in overall cellular excitability. Although multiple physiologic mechanisms may lead to an effective increase in conductance at the soma (see Discussion), for computational simplicity, we increased the background conductance in the model by adjusting only the rates of balanced (excitatory and inhibitory) background synaptic inputs. In the CA model, background synaptic input rates were tenfold higher than those in the sham model. Although such a rate increase is clearly not physiologic, the overall increase in conductance may reflect a combination of multiple physiologic factors, e.g., synaptic rates, peak synaptic currents, and spine remodeling (see Discussion). Both excitatory and inhibitory background rates were increased by the same factor, which maintains a relative balance of opposing synaptic forces while increasing conductance variability. Reflecting the unchanged excitatory–inhibitory balance and consistent with experimental data, spontaneous firing rates in the CA and sham models remained similar (see Figs. 4 and 5 below).

In the CA model, increased background conductance decreases the mean depolarization evoked by incoming synaptic inputs. When spike thresholds are removed, the slope of mean depolarization as a function of synaptic input rate was shallower in the CA model than in the sham model (Fig. 4*A*). Similarly, voltage change in response to a single incoming synaptic input was smaller in the CA than in the sham model (Fig 4*B*). The ability of a neuron to sum synaptic currents is determined by its input resistance, which, in turn, is inversely proportional to net conductance (Softky and Koch, 1993). In the CA model, increased conductance decreased input resistance. Decreased input resistance diminished the ability of CA neurons to integrate incoming synaptic inputs above action potential threshold. A similar reduction in stimulus-evoked potentials was observed by measuring field potentials in a model of neonatal hypoxic ischemia (Quairiaux et al., 2010).

When spike thresholds are reintroduced into the models, the CA model reproduces preserved AW-evoked and reduced PW-evoked firing rates observed *in vivo*. The spiking models were tested at different thalamocortically derived synaptic input rates, resulting in output rates that were comparable to spontaneous, AW-evoked, and PW-evoked firing rates. At synaptic input rates simulating spontaneous firing, both CA and sham models generated APs at comparable frequencies (Fig. 4*C*, top row). Similarly, at synaptic input rates simulating AW-evoked firing, CA and sham models generated similar output firing rates (Fig. 4*C*, middle). However, at higher input rates mimicking PW stimulation, the CA model generates APs at a lower frequency than the sham model (Fig. 4*C*, bottom row).

To understand how AP threshold and subthreshold synaptic mechanisms interact to give differential responses to whisker stimuli, we examined the input–output transfer function (Fig. 5). The F-I curve (firing rate vs. input) quantifies the level of output as a function of input amplitude. An F-I curve with a larger slope (higher gain) reflects a neuron whose output firing rate is more sensitive to incremental increases in the amplitude of synaptic inputs. Background synaptic conductance (i.e., stimulus input-independent conductance) fluctuations can affect the output firing rate by modulating the magnitude of voltage fluctuations and modulating the mean membrane voltage time scale. Through the action of both of these mechanisms, the changes in the background synaptic conductance state can modulate the gain of firing rate transfer functions (Chance et al., 2002; Mitchell and Silver, 2003). Different transfer functions, effectively modulated by background activity, differentially affect spike output to whisker-evoked stimuli in sham and CA conditions.

**Figure 6.**
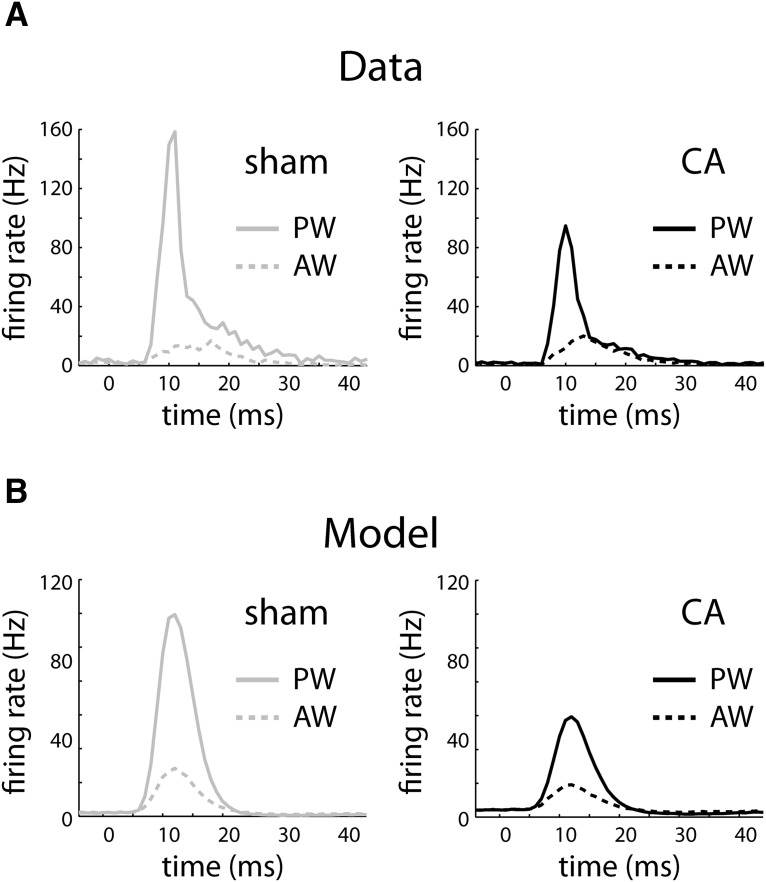
Peristimulus time histograms (PSTHs) of real and model neurons in response to PW and AW whisker deflections. ***A***, Average PSTH of L4 neurons from sham rats in response to a whisker deflection beginning at 0 ms. The PW PSTH rapidly increases to high instantaneous firing rates and then relaxes more slowly to spontaneous rates (solid gray), whereas the AW PSTH peaks at smaller values (dashed gray). In contrast, the average PW PSTH peaks at a smaller rate (solid black), relative to sham neurons, whereas the AW PSTH (dashed black) peaks at similar values to the sham neurons. PSTHs are calculated using 1-ms bins. Instantaneous firing rates are calculated by dividing the observed counts by 1 ms. ***B***, Sham and CA model neurons reproduce the relative PW and AW PSTH responses. The sham PW PSTH (solid gray) peaks at a higher instantaneous firing rate relative to the CA model (solid black), whereas the sham (dashed gray) and CA (dashed black) AW PSTHs peak at similar values.

The gain of the CA model is lower than that of the sham model, leading to decreased responses to larger input stimuli (Fig. 5). The time course of the simulated thalamocortical inputs is shown along the bottom of the F-I curve. The time scales of both AW and PW thalamocortical inputs were set to the same value, and the ratio of their amplitudes (area under the TC input curves, bottom of Fig. 5) was set to 0.4 based on previous observations (Shoykhet and Simons, 2008). Spontaneous and AW-evoked input rates led to output rates that were comparable in both the CA and sham models. PW stimulation led to a lower output-firing rate in the CA model because of reduced gain resulting from increased balanced (excitation and inhibition) background synaptic conductance. When we simulated the time-dependent model (Eqs. 3–7), both the sham and the CA models produced PW and AW responses (Fig. 6*B*) qualitatively similar to those seen in the data (Fig. 6*A*). Quantitatively, the sham model produced an AW/PW ratio of 0.24, whereas the CA model produced an AW/PW ratio of 0.46. These simulated results reproduced the difference between AW/PW ratios observed in L4 RSUs in sham (0.23 ± 0.03) versus CA (0.49 ± 0.03) rats *in vivo*.

We set the parameters of our models based in responses to stimulus onsets (ON). To test our model further, we examined how well the models simulate responses to stimulus offsets (OFF), a stimulus that evokes responses different from stimulus onsets in sham and CA neurons. Normally, in L4 barrel RSUs, OFF responses are smaller than ON responses, but the difference is not as great as that between AW and PW responses (Simons and Carvell, 1989). Furthermore, OFF/ON ratios in barrel RSUs are smaller than those in VPm neurons, reflecting the effects of local circuitry (Simons and Carvell, 1989; Shoykhet et al., 2012). Experimentally observed OFF/ON ratios of L4 RSUs in sham rats (0.71 ± 0.06) were comparable to those of L4 RSUs in CA rats (0.67 ± 0.06) and smaller than those of VPm neurons. We tested how well our model predicts the differences between L4 RSU OFF and ON responses by driving the model with two firing rates whose amplitude ratio is 0.8, approximating the OFF/ON ratio of VPm neurons in both sham and CA rats (Shoykhet et al., 2012). No other changes were made to model parameters. The resulting OFF/ON ratio was 0.65 in the sham model and 0.69 in the CA model. These simulated OFF/ON ratios fell within the SEs of experimentally observed values, suggesting that our model, developed initially to account for PW and AW responses, similarly approximates ON and OFF responses in sham and in CA animals.

### Correlation of neural activity in sham and CA rats

**Figure 7.**
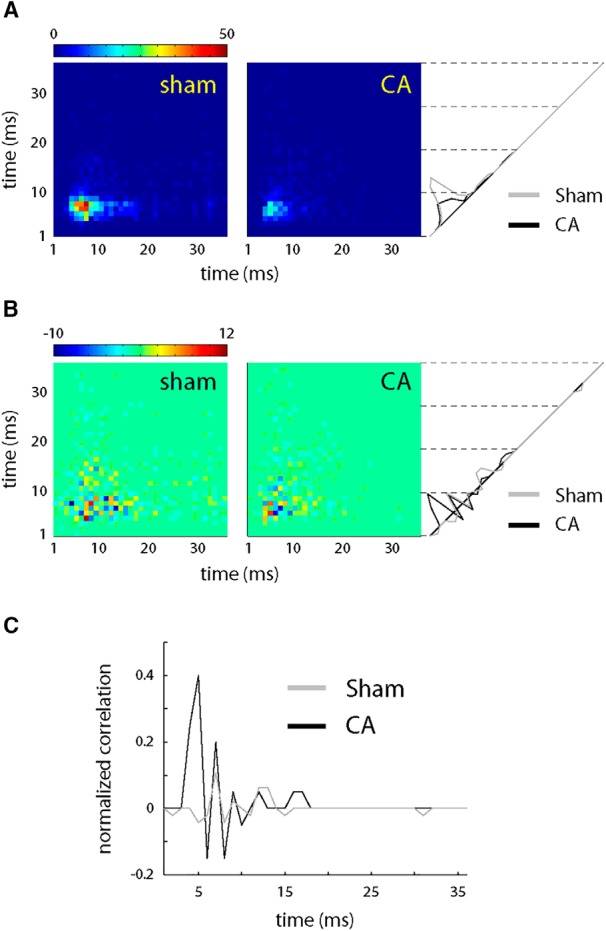
Pairwise correlation structure in sham and CA L4 RSUs. ***A***, The average joint peristimulus time histogram (jPSTH) of pairs of L4 excitatory neurons reveals a robust coactivation in response to whisker deflection in sham neurons (left). CA neuron coincident firing is smaller, as revealed by a lower-magnitude jPSTH (right). Comparing the projections of the jPSTHs along the diagonal gives the temporal profile of the cross-correlation at lag zero. ***B***, Shuffle-corrected jPSTHs (see Materials and Methods) reveal higher levels of coincident firing in CA versus sham RSUs. ***C***, Normalizing the cross-correlation from the shuffle-corrected jPSTHs by the peak value of the raw jPSTHs (see Materials and Methods) reveals a larger relative correlation for L4 RSU in CA rats.

Correlation of neural activity likely plays an important role in functionally connecting different brain regions (Fries, 2005) and efficiently activating postsynaptic targets by synchronous presynaptic neural populations (Bruno and Sakmann, 2006). However, excessive neural correlation can be detrimental for stimulus coding and discrimination (Averbeck et al., 2006), and active mechanisms normally maintain relatively low stimulus-induced correlations among cortical cells (Ecker et al., 2010; Renart et al., 2010; Ly et al., 2012; Middleton et al., 2012). To examine correlations in sensory stimulation-evoked neuronal activity, we plotted joint PSTHs (jPSTHs; see Materials and Methods) of simultaneously recorded responses of L4 RSUs to PW deflections. The peak of the raw jPSTH for jointly recorded CA neurons was smaller than the peak of the jPSTH for sham neurons (Fig. 7*A*), consistent with smaller PW-evoked responses in CA versus sham rats (Fig. 3). The shuffle-corrected jPSTH, which more closely reflects within-network rather than externally driven stimulus-dependent correlations, revealed higher levels of above-chance coincidence in CA neurons compared with sham (Fig. 7*B*). Because coincidence of two Poisson processes increases as their frequency increases even if the two processes are entirely independent, we further normalized the diagonal of the shuffle-corrected jPSTH by the product of individual PSTHs to get true correlation. The normalized, shuffle-corrected jPSTH in Fig. 7*C* still showed increased correlation among L4 RSUs in CA compared with sham rats. Such increases are likely to lead to poorer discrimination among stimuli in CA animals (see Discussion).

**Figure 8.**
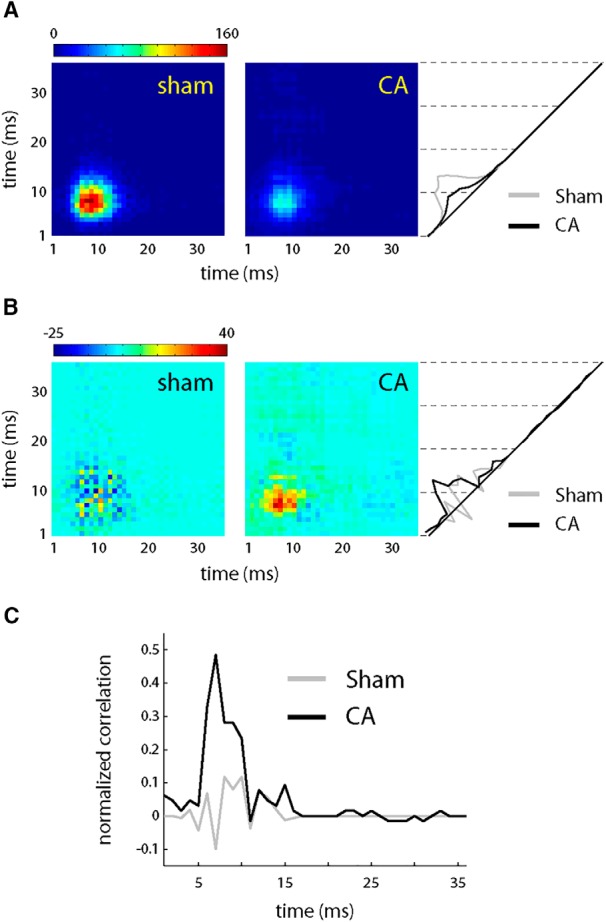
Pairwise correlation structure in sham and CA model L4 RSUs. ***A***, The average jPSTH of pairs of L4 model neurons reveals robust coactivation in response to whisker deflection in sham neurons (left). CA neuron coincident firing is smaller in magnitude, as revealed by a lower-magnitude jPSTH (right). Comparing the projections of the jPSTHs along the diagonal gives the temporal profile of the cross-correlation at lag zero. ***B***, Subtracting the shuffle-corrected jPSTHs (see Materials and Methods) reveals more similar sham and CA jPSTH structure. ***C***, The normalized cross-correlation reveals a larger relative correlation for CA model neurons that is qualitatively consistent with the experimental data in Fig. 7.

To better understand the potential mechanism of increased correlation in PW-evoked spiking among CA RSUs, we simulated joint activity of pairs of neurons. We made a fraction of excitatory and inhibitory synaptic inputs common to both model neurons using a correlation coefficient, *c* (see Materials and Methods). We set *c* = 0.15, a value consistent with low correlation among L4 RSUs observed experimentally (Khatri et al., 2009). All other parameters in the model remained unchanged. Raw, shuffle-corrected, and normalized jPSTHs for model neurons (Fig. 8) were in good agreement with experimental data (Fig. 7). Thus, high correlation in synaptic inputs leads to greater correlations in firing among CA RSUs versus sham RSUs.

## Discussion

In this study, we examined how cardiac arrest during development impacts the function of cortical circuits in adulthood. We found that months after initial injury, responses to principal whisker deflections were smaller in magnitude in CA animals compared with sham rats, whereas responses to adjacent whisker deflections were comparable in the two groups. A disproportionate decrease in PW-evoked responses leads to broadening of receptive fields among L4 barrel RSUs. Mathematical modeling suggests that increased background conductance of individual neurons, without any other alterations in cellular function or synaptic circuitry, can account for the observed broadening of receptive fields. We also found that spiking activity of barrel neurons was more correlated in CA animals compared with sham rats, indicating more synchronous firing in CA survivors. Together, *in vivo* and modeling data suggest that CA and resuscitation during development permanently affect cortical circuit function in survivors.

### Cortical circuit function after a hypoxic-ischemic insult

Receptive field broadening effected by decreased magnitude of most robust responses may be a general feature of cortical circuit dysfunction after hypoxic-ischemic injury. In an experimental model of neonatal hypoxia-ischemia in 3-d-old mice, epicranial mapping during recovery revealed depressed sensory responses in the primary somatosensory cortex (Quairiaux et al., 2010). Similarly, in the mouse visual system, responses in the primary visual cortex are reduced in survivors of neonatal hypoxia-ischemia (Failor et al., 2010), and neurons in the primary auditory cortex display reduced response amplitudes and broadened tuning curves in rats subjected to two 12-min-long periods of asphyxia shortly after birth (Strata et al., 2010). These studies, encompassing multiple sensory modalities, and our current results show that hypoxic-ischemic insults result in less responsive and more broadly tuned cortical circuits.

### Potential mechanisms of cortical circuit dysfunction after CA

Our mathematical modeling indicates that an increase in background synaptic conductance of barrel RSUs can account for both smaller PW-evoked responses and larger AW/PW ratios in RSUs of CA survivors. Prior combined experimental/modeling studies demonstrate how changes in background synaptic conductance modulate response gain (Chance et al., 2002). Balanced increases in background excitatory and inhibitory conductances increase membrane voltage variability, decrease input resistance, and shorten membrane time constants, effectively shunting external synaptic inputs (Bernander et al., 1991). Together, these effects shunt synaptic inputs, reducing the slope of input-output firing curve and decreasing the gain. The decrease in gain, in turn, disproportionately reduces responses to stronger stimuli, e.g., PW-evoked deflections. Hence, cortical neurons in a high-conductance state integrate spatiotemporal information differently than neurons with less active background synaptic activity (Destexhe et al., 2003).

**Figure 9.**
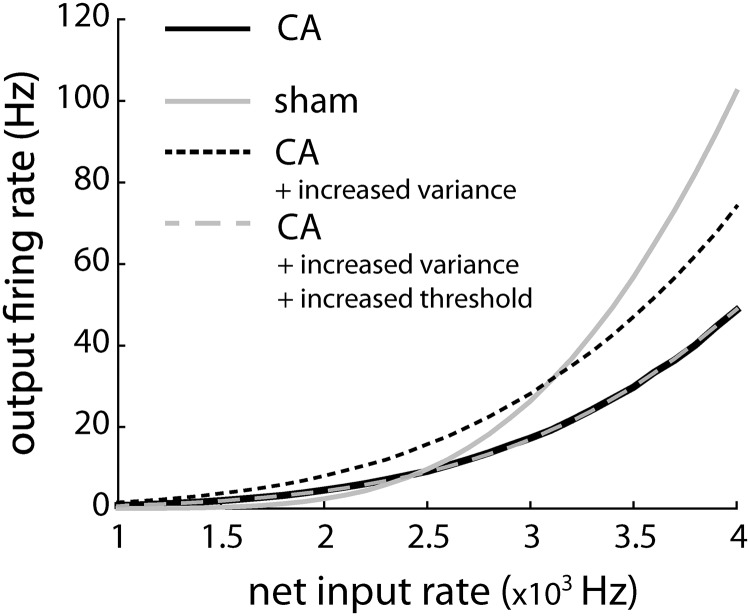
Alternate mechanisms of gain control in CA model neurons. The original sham and CA model from Fig. 5 are shown in solid gray and black curves. The dashed black line shows increased gain of the F-I curve when the maximum amplitude of a unitary background input, instead of the net background conductance event rate as in the original CA model, is increased. The overall firing rate, however, is also increased, which is inconsistent with experimental data. Decreasing the mean bias current slightly shifts the F-I curve (gray dashes) downward to match the data and the original CA model.

Animal models of global ischemia have uncovered a number of anatomic and synaptic changes during postinjury periods. Among these changes are a transient change in spine density, persistent changes in the distribution of dendritic branching points (Ruan et al., 2007), and the relative prevalence of different spine morphologies (Ruan et al., 2009). To show that different gain control mechanisms can lead to the same results that we observe experimentally, we simulated an injury model in which the increased net excitation and inhibition were implemented as an increase in the peak conductance of individual synaptic events. The overall rate of presynaptic input remained the same. By increasing the maximal conductance, we observed a decreased gain but overall higher firing rate of the CA model output (Fig. 9, black dashed line). With an additional decrease in the bias current driving the model (13% decrease), we were able to get quantitative agreement of the F-I curve with that of the original CA model (Fig. 9, gray dashed line). These findings highlight that there are potentially different physiologic mechanisms of implementing gain control by altering background inhibition and excitation in a concerted manner. However, these results, together with the results of Fig. 6, support the hypothesis that alterations in both excitatory and inhibitory circuitry in the cortex underlie reduced PW-driven responses and less well-defined whisker receptive fields in CA survivors.

Decreased effective inhibition alone cannot account for our results. For example, sensory deprivation during development, followed by a period of whisker regrowth, leads to increased AW/PW ratios of L4 RSUs via doubling of connection probability between VPm neurons and RSUs (Simons et al., 2015) and effective net decrease in intrabarrel inhibition (Shoykhet et al., 2005). The effect of sensory deprivation, however, is to increase AW-evoked responses out of proportion to the PW-evoked responses, which leads to increased AW/PW ratios via a mechanism distinctly different from that observed in CA survivors. Consistent with population models (Kyriazi and Simons, 1993; Pinto et al., 2003) and *in vivo* data (Kyriazi et al., 1996), reduced inhibition in our model increased AW-evoked firing rates out of proportion to the PW-evoked rates (data not shown). Thus, reducing intracortical inhibition alone increases AW/PW ratios, but it cannot explain the decreased PW-evoked and preserved AW-evoked response magnitudes we observed in CA survivors.

The finding of increased synchrony among L4 RSUs in CA survivors lends further support to the hypothesis that background synaptic conductances are increased in the barrel circuit after CA. Modeling data indicate that introducing an identical degree of correlation into synaptic inputs results in higher correlation among RSUs in the CA circuit than in the sham circuit. Input synaptic correlation was introduced in the model through background conductance. In the CA model, background conductance represents a larger proportion of the entire membrane conductance. As a result, the relative input correlation is higher in the CA model than in the sham model. Higher relative input correlation is transferred to the output function, resulting in higher spiking correlation in the CA model. The ability of the model to reproduce experimentally determined firing statistics of simultaneously recorded L4 RSUs in CA and sham rats suggests that a balanced increase in background excitatory and inhibitory conductances may indeed be the mechanism underlying functional changes in the barrel circuit of cardiac arrest survivors. This hypothesis can be tested experimentally using *in vivo* intracellular recordings (Wilent and Contreras, 2005).

### Behavioral implications of broadened receptive fields and increased spiking coherence

Broadened receptive fields of barrel RSUs in CA survivors will likely degrade the barrel circuit’s capacity to differentiate between sensory stimuli arising from individual whiskers. In population coding, well-tuned neurons with small receptive fields generally outperform broadly-tuned neurons with large receptive fields (Zhang and Sejnowski, 1999; Averbeck et al., 2006). Behaviorally, enlarged barrel RSU receptive fields are associated with permanently degraded texture-discrimination capacity in rats deprived of normal sensory input during development (Carvell and Simons, 1996). Although the mechanism of receptive field broadening likely differs between sensory-deprived animals and CA survivors, the detrimental impact on whisker-barrel system function is likely to be similar.

Increased coherence among barrel RSUs may also affect whisker-based behaviors. Coherence among neurons in a given population may increase the probability of stimulus detection in that population’s postsynaptic target (Salinas and Sejnowski, 2001; Middleton et al., 2009). Thus, increased coherence among barrel RSUs may be a beneficial adaptation that compensates for reduced firing rates in CA survivors and improves stimulus detection. The price for this benefit, however, may be reduced stimulus discrimination (Wang et al., 2010). Modeling studies suggest that the detrimental impact of increased coherence on neuronal population coding increases with the size of the population: specifically, increased coherence leads to a decrease in stimulus discriminability from population responses (Averbeck et al., 2006). Each L4 barrel contains 4000–5000 neurons (Meyer et al., 2010). In a neuronal population of this size, increased coherence can lead to a substantial decrease in discriminability among sensory input signals (Averbeck et al., 2006). Thus, increased coherence among barrel RSUs in CA survivors may further decrease the stimulus coding capacity of the barrel circuit. Whisker-based object detection and texture discrimination may thus represent sensitive behavioral tests of putative therapeutic strategies in cardiac arrest survivors.

## Synthesis

Reviewing Editor: Jun Chen, University of Pittsburgh Decisions are customarily a result of the Reviewing Editor and the peer reviewers coming together and discussing their recommendations until a consensus is reached. When revisions are invited, a fact-based synthesis statement explaining their decision and outlining what is needed to prepare a revision will be listed below. The following reviewer(s) agreed to reveal their identity: Pelin Cengiz, Shan Ping Yu

The authors have studied the effects of neonatal cardiac arrest on the developing brain and network function in adulthood. They have found that sensory whisker responses are diminished and neurons have broader receptive fields, when compared to shams. Also, the spiking activity is more correlated which would behaviorally manifest as reduced sensory discrimination, as presented in discussion. Authors have used mathematical modeling to posit increased background conductance of individual neurons that explains their experimental data. The work is important as it puts forward a new mechanism that may underlie abnormal network function after hypoxic ischemic brain injury in a developing brain. I have following comments:

• Please show some example RSU waveforms and inter-spike interval histograms. There is a paucity of details on neurophysiologic recordings- what was the quality of units that were analyzed, details on spike sorting, electrode array used, etc.

• Is there a reason for restricting analyses to regular spiking (RS) cells? Is similar phenomena seen for fast spiking (FS) interneurons, such as diminished principal whisker (PW) response, more correlated firing, etc? The authors have increased both excitatory and inhibitory background rates by the same factor in their computational model; it would be nice to see experimental data of a few FS cells.

• Ln 84: “...frank cell death” sentence could be rephrased

• Ln 102: justification for using only female rats should be expanded. Please specify if neonatal cardiac arrest is more prevalent in either gender

• Ln 456 : Fig 5 is referenced in text prior to Fig 4, this should be rearranged
